# Validation of the Dutch version of the Multidimensional Adolescent Functioning Scale (MAFS)

**DOI:** 10.1186/s12955-020-01517-7

**Published:** 2020-09-17

**Authors:** Sally A. Mayle, Jessica M. de Klerk-Sluis, Ashleigh Lin, Alison R. Yung, Klaas J. Wardenaar, Sanne R J Broekman, W Heleen Pluim, Johanna T. W. Wigman

**Affiliations:** 1 Interdisciplinary Centre for Psychopathology and Emotion regulation, University Medical Center Groningen, Groningen, The Netherlands; 2 Telethon Kids Institute, the University of Western Australia, West Perth, Australia; 3 School of Health Sciences, University of Manchester, Manchester, UK; 4 Greater Manchester Mental Health NHS Foundation Trust, Manchester, UK; 5 Centre for Youth Mental Health, University of Melbourne, Melbourne, Australia; 6 Faculty of Medical Sciences, Academic Centre of Psychiatry, Hanzeplein 1, 9713 GZ Groningen, the Netherlands

**Keywords:** Multidimensional adolescent functioning scale, MAFS, Adolescents, Functioning, Psychosocial, Paediatric, Scale

## Abstract

**Background:**

The Multidimensional Adolescent Functioning Scale (MAFS) is a 23-item, self-report questionnaire assessing psychosocial functioning in adolescents aged 12–17 years. It captures three domains of functioning: ‘general functioning’, ‘family-related functioning’, and ‘peer-related functioning’. The original English version has good psychometric properties. The aim of the current paper was to translate the MAFS to Dutch and to investigate the psychometric properties of this translation.

**Methods:**

After translation, the Dutch MAFS was assessed in 397 adolescents aged 12–17 years, assessed at schools. Internal consistency, factor structure and correlations with other questionnaires assessing functioning, psychopathology and well-being were investigated.

**Results:**

A hierarchical/bifactor model with a general factor that loads on all items (MAFS-general) and three group factors, loading respectively on the GF, FF and PF items, was found to describe the data best. Internal consistency of the MAFS total score (α = 0.87) was good and of the subscales (α = 0.74–0.80) acceptable. Comparable alphas were found in males and females. Correlations between MAFS subscales ranged from 0.33 to 0.43, indicating sufficient differentiation. The MAFS general factor score and group factor scores showed positive correlations with other measures of good functioning and well-being, and negative correlations with measures of psychopathology, supporting convergent and divergent validity.

**Conclusions:**

The Dutch translation of the MAFS has adequate psychometric properties to assess three domains of functioning in adolescents from the general population aged 12–17 years. The MAFS is freely accessible in the Appendix and easy to administer.

## Introduction

Psychosocial functioning is an important concept to measure as it is closely related to mental health and mental illness [1] while at the same time partly independent of mental health status [2]. Psychosocial functioning is a multidimensional construct, encompassing different domains such as social or occupational functioning. The nature of the construct of functioning may change during the life course, as different domains play various roles with advancing age. Adolescence, for example, is a period of major biological and psychosocial change [3] and, although functioning among family remains crucial [4], functioning within the peer group becomes increasingly important. During adolescence, the process of individualization begins as adolescents become more separated from their parents [5]. This may result in differentiated levels of peer and family functioning. Distinct domains of psychosocial functioning have also been shown to be differentially related to several important health outcomes in adolescents. Studies have shown that adolescents who perceive more negative family interactions have a higher risk of developing depression [6, 7] and sexually transmitted diseases [8], whereas positive family functioning was associated with healthier lifestyle [9]. Functioning in the context of peers is also related to psychiatric and physical outcomes. Suicidal ideation, for example, has been found to be more severe among adolescents who feel rejected by or receive less support from their peers [7]. Adolescents who function well among peers show behaviour associated with a healthier lifestyle (such as a healthy diet and more exercise) compared to those with poor peer functioning [10].

Despite the importance of psychosocial functioning in adolescence, there are a lack of useful tools to reliably measure it. Most validated instruments developed to assess psychosocial functioning are designed either for adults (e.g. the Social and Occupational Functioning Assessment Scale (SOFAS) [11];), which makes them less applicable to adolescents [12]. When designed for children and young people, they focus primarily on clinical populations [13]. To fill this gap, the *Multidimensional Adolescent Functioning Scale* (MAFS) has been developed [2]. Researchers and clinicians from an Australian research group together identified the lack of appropriate instruments for measuring adolescent functioning and collaborated on designing a list of items to capture this. This list was shortened, and this shortened version was validated and eventually published as the MAFS questionnaire [2]. The MAFS is a 23-item, self-report questionnaire assessing psychosocial functioning. It has two important advantages over existing measures. First, it was specifically developed to measure *adolescent* functioning and, as such, is applicable to young people between the ages of 12 and 17 years. It captures three domains of functioning that are important in the adolescent period, namely ‘general functioning’, ‘family-related functioning’, and ‘peer-related functioning’. Second, the MAFS takes *good* psychosocial functioning as a point of reference and does not assess psychopathological symptoms, which makes it useful to measure variations in functioning levels in non-clinical settings. Compared to adolescents from a clinical population, functional changes in adolescents from the general population are likely to more subtle. Instruments that take pathological functioning as a reference lack sensitivity to small changes [12] which might still be indicative of psychological distress and increased risk for future psychopathology. In addition, the assumption that healthy individuals all function in the same way can be challenged [1], as even within well-functioning individuals, a more nuanced profile of the level and nature of functioning can be informative.

Although the MAFS has been used as an outcome measure in several studies (e.g., [14, 15]), only the English version of the MAFS has been validated at present. This version was found to have good psychometric properties in adolescents [2]. In this study, we translated the MAFS into Dutch with the aim of investigating the psychometric properties of this translation in a sample of adolescents from the general population of the Netherlands.

## Methods

### Participants

At the start of the study, 29 schools in the northern part of the Netherlands were approached for participation. These schools were chosen based on convenience in terms of geographical location and previous contacts between the schools and the researchers. Of these, 9 agreed to participate, 6 did not respond, and 14 declined participation. Inclusion criteria were an age in the range of 12–17 years, attending school on the day of assessment and ability to speak and write Dutch. Schools for specialized education were not approached for participation. No other exclusion criteria were used.

In total, 397 adolescents aged 12 to 17 years were recruited between February and May 2017. In the Netherlands, secondary education starts at age 12 and works according to a differentiated, multi-track system. Students from these tracks were grouped into three levels of education: low (Dutch: VMBO, preparing for secondary vocational education), middle (Dutch: HAVO, preparing for higher professional education) and high (Dutch: VWO, preparing for university education).

### Procedure

Questionnaires were administered during school hours in a classroom setting. Information about the purpose of this study and instructions were in most cases (*n* = 335) given directly by the researchers. In some cases (*n* = 62), the teachers were instructed beforehand by the researchers and then they provided the information and instructions to the students. Written informed consent was obtained from all participants. Written informed consent for adolescents between 12 and 15 years of age were obtained from both the adolescent and one from their parent or guardian. The questionnaires were administered anonymously. It took approximately twenty-five minutes to complete the questionnaires and participants had the opportunity to enter in a draw with the chance of winning a prize. The study protocol was reviewed and approved by the local internal ethical committee.

### Instruments

The Multidimensional Adolescent Functioning Scale (MAFS) is a 23-item, self-report questionnaire assessing psychosocial functioning of adolescents between age 12 and 17 years [2]. The questionnaire consists of three subscales, ‘general functioning’ (MAFS-GF, ten items), ‘family-related functioning’ (MAFS-FF, seven items), and ‘peer-related functioning’ (MAFS-PF, six items). Scores per subscale as well as a total score (MAFS-TS) were computed. Questions were answered on a 4-point Likert scale ranging from ‘not at all’/‘rarely’ to ‘always’/‘almost always’. On the majority of items, a higher score indicates better functioning, but there are also five reverse-scored items (lower score indicates better functioning: items 8, 9, 13, 15 and 18) that need to be rescored before scale-score computation. On each item, participants could also answer ‘not applicable’. In analyses of the item-level data, ‘not applicable’ responses were treated as missing values. Higher scores on the MAFS scales indicates better overall functioning.

The MAFS was translated to Dutch by authors of the manuscript (SM, JS, HP and SB); this translation was reviewed by the research supervisor (JTWW). After adjustments, the Dutch version of the MAFS was translated back to English by a professional translator who was blind to the original English version. This back-translated version was reviewed by the developers of the original MAFS (AY and AL). In consultation with the original authors (AY and AL), minor adjustments were made.

The Strengths and Difficulties Questionnaire (SDQ, [12]) is a 25-item self-report questionnaire widely used to measure emotional and behavioral problems in children and adolescents. It has five subscales: ‘Emotional symptoms’, ‘Conduct problems’, ‘Hyperactivity/inattention’, ‘Peer problems’, and ‘Prosocial behavior’. Questions were answered on a 3-point Likert scale ranging from ‘not true’ to ‘certainly true’. The first four subscales are summed to calculate a Difficulties score, indicating psychopathology. The subscale ‘Prosocial behavior’ indicates good functioning.

The Dutch Groningse Vragenlijst Sociaal Gedrag (Groningen Questionnaire for Social Behavior [GVSG], [16]) measures self-reported social functioning of adults. It consists of 40 items subdivided into eight subscales: ‘Parents’, ‘Friends’, ‘Education’, ‘Household’, ‘Work’, ‘Leisure time’, ‘Intimate relationships’ and ‘Children’. We only used the subscales ‘Parents’ and ‘Friends’ because these were the most relevant to our study. Questions were answered on a 4-point Likert scale ranging from ‘never’ to ‘always’. Higher scores indicated better functioning.

The WHO Well-being Index [17] consists of five questions about emotional well-being. The questions are scored on a six-point Likert scale ranging from ‘not at all’ to ‘constantly’. Higher scores indicate higher levels of well-being.

Additional questions were included to assess demographic variables: gender, age and level of education (divided into lower, middle and higher secondary education levels). In addition, to gain insight into day-to-day functioning, one question about the number of days that the adolescent had missed school due to illness and one question about the average school grade of the participant were included. In the Dutch school system, school grade can range from 1 (lowest) - 10 (highest). Finally, participants were asked to rate their general happiness on a scale from 1 to 10.

### Statistical analyses

Analyses were performed to investigate the psychometric properties of the translated version of the MAFS, of which most analyses were done for the total sample (*n* = 397) and some analyses for males and females separately. Because there were some missing responses on the MAFS, as many analyses as possible were run using a full information maximum likelihood estimation framework (FIML; see below). For calculations involving raw item scores, pairwise complete observations were used (e.g., for internal consistency).

First, the sample characteristics were investigated and compared between male and female participants using Chi-square tests to compare proportions on categorical outcomes and independent samples t-tests to compare means on continuous outcomes. In case of non-normally distributed variables, a non-parametric (Mann-Witney U) test was used for comparison.

Second, we used confirmatory factor analysis (CFA) to test the fit of the previously reported latent 3-factor structure (MAFS-GF, MAFS-FF, and MAFS-PF) and compared it to a 1-factor model (MAFS-Total) and a hierarchical/bifactor model [18] with four uncorrelated factors: a general factor loading on all items (MAFS-general) and 3 group factors, loading respectively on the GF, FF and PF items. In the CFA models, one loading per factor was fixed to 1 to set the scale of the model. Because the MAFS contains reverse-scored items, potential method effects on model fit were investigated by fitting each tested model a second time, while freely estimating the correlations between the reverse-scored items’ residuals. For pragmatic reasons we used two estimators. First, model estimation was done using robust maximum likelihood estimation (MLR), which is suitable for categorical data and handles missing data by FIML. The models were compared on the Akaike Information Criterion (AIC) and Bayesian Information Criterion (BIC), with the lowest values indicating the model that best describes the data. Second, the same models were also estimated using mean and variance adjusted weighted least squares (WLSMV) estimation for categorical data. To handle missing data, this approach fits the models on pairwise estimated polychoric correlation matrices, which is efficient but less consistent than FIML. However, WLSMV was needed to estimate the models with correlated residuals (which is very hard using MLR) and to obtain additional measures of fit (Comparative Fit Index and Root Mean Error of Approximation (RMSEA)). A CFI > 0.95 and an RMSEA< 0.06 were considered to indicate good fit. Because the lowest response category was endorsed rarely on MAFS items, the lowest two categories were merged for the factor analyses to prevent computational issues.

After identification of the optimal model, associations of demographic factors and other psychometric measures with the factor scores were investigated. In the interpretation of the results, positive regression coefficients for the GVSG, the SDQ Prosocial behavior score, well-being and happiness were interpreted as signs of convergent validity and negative coefficients for the SDQ Difficulties score were interpreted as supportive of divergent validity. In addition, to evaluate associations between MAFS factors and day-to-day functioning, correlations with school grades and number of sick days during the past 4 weeks were calculated. Additionally, to gain a complete insight into the scales’ validity, correlations between the MAFS raw subscale sum scores and the other measures were calculated.

To gain insight into the internal consistencies of the MAFS scale sum scores, the Polychoric Ordinal Alpha (α) was calculated, which provides a reliable estimation than the regular Cronbach’s Alpha, which is better suited to continuous items [19]. An α of 0.70–0.80 was considered as acceptable, of 0.80–0.90 as good, and ≥ 0.90 as excellent [20]. Furthermore, Spearman correlations were calculated between the raw MAFS subscales to investigate the level of interrelatedness of the subscales.

*P*-values < 0.05 were considered statistically significant. Polychoric Ordinal Alpha was computed using functions from the ‘psych’ package [21] and CFA was run using Mplus v 5.0. All other analyses were performed in R [22].

## Results

### Sample characteristics and missing data

The sample characteristics are shown in Table 1. The mean age was 15.2 years and there were twice as many females as males. The majority followed a higher level of education. Of the total sample, 95 (23.9%) had one or more missing values on the MAFS, which could be a missed item or a response of ‘not applicable’. Of these, 27 participants missed one or more items and 69 endorsed ‘not applicable’ on one or more items of the MAFS. The 95 participants with missing values did not differ from the rest of the sample in terms of gender distribution (χ^2^ = 0.04; *p* = 0.84) and age (t = 0.90; *p* = 0.37), but they were more often in the low and middle education level groups (30.4 and 33.8%, respectively) than in the high education level group (18.8%; χ^2^ = 9.53; *p* = 0.009). Similar associations were found when comparing the 69 participants that endorsed ‘not applicable’ with the participants that did not endorse this response. No differences were found in the demographics between the 27 participants with a ‘regular’ missing value and the rest of the sample that did not have regular missing values.

**Table 1:** Sample characteristics and gender comparisons

Variables	Total sample (*n* = 297)		Male sample (*n* = 133)		Female sample (*n* = 264)		*p-*value for gender comparison	Type of test
Education (low/medium); n & %	78.0	19.6%	14.0	10.5%	64.0	24.2%	0.005	Chi-square
Education (Middle); n & %	74.0	18.6%	27.0	20.3%	47.0	17.8%	–	–
Education (High); n & %	245.0	61.7%	37.0	27.8%	85.0	32.2%	–	–
Age in years (mean & sd)	15.2	1.7	15.2	1.7	15.2	1.7	0.74	t-test
MAFS Total (mean & sd)	79.0	6.7	77.9	6.6	79.5	6.7	0.05	t-test
MAFS Family (mean & sd)	25.5	2.5	25.4	2.4	25.6	2.6	0.53	t-test
MAFS Peer (mean & sd)	20.2	2.5	19.6	2.6	20.6	2.4	< 0.001	t-test
MAFS General (mean & sd)	32.9	3.6	32.7	3.6	33.1	3.7	0.36	t-test
GVSG Total (mean & sd)	100.4	8.0	98.5	7.2	101.3	8.2	0.06	t-test
GVSG Parents (mean & sd)	17.2	2.6	17.1	2.5	17.3	2.7	0.52	t-test
GVSG Friends (mean & sd)	17.5	2.3	16.8	2.3	17.9	2.3	< 0.001	t-test
SDQ Difficulties (mean & sd)	10.6	4.8	10.3	4.5	10.7	5.0	0.46	t-test
SDQ Prosocial (mean & sd)	8.3	1.6	7.6	1.8	8.6	1.3	< 0.001	t-test
WHO total (mean & sd)	19.7	4.7	20.4	4.3	19.4	4.8	0.04	t-test
Sick days (median & IQR)	0.0	1.0	0.0	1.0	0.0	2.0	0.11	M-W U-test
School grade (mean & sd)	7.0	0.7	6.8	0.7	7.0	0.7	< 0.001	t-test
Happiness (mean & sd)	7.5	1.3	7.7	1.4	7.4	1.3	0.11	t-test

MAFS = Multidimensional Adolescent Functioning Scale, GVSG = Groningse Vragenlijst Sociaal Gedrag, SDQ = Strengths and Difficulties Questionnaire, WHO = WHO Well-being Index, sd = standard deviation; M-W U-test = Mann-Whitney U-test

### Confirmatory factor analysis

**Fig. 1:**
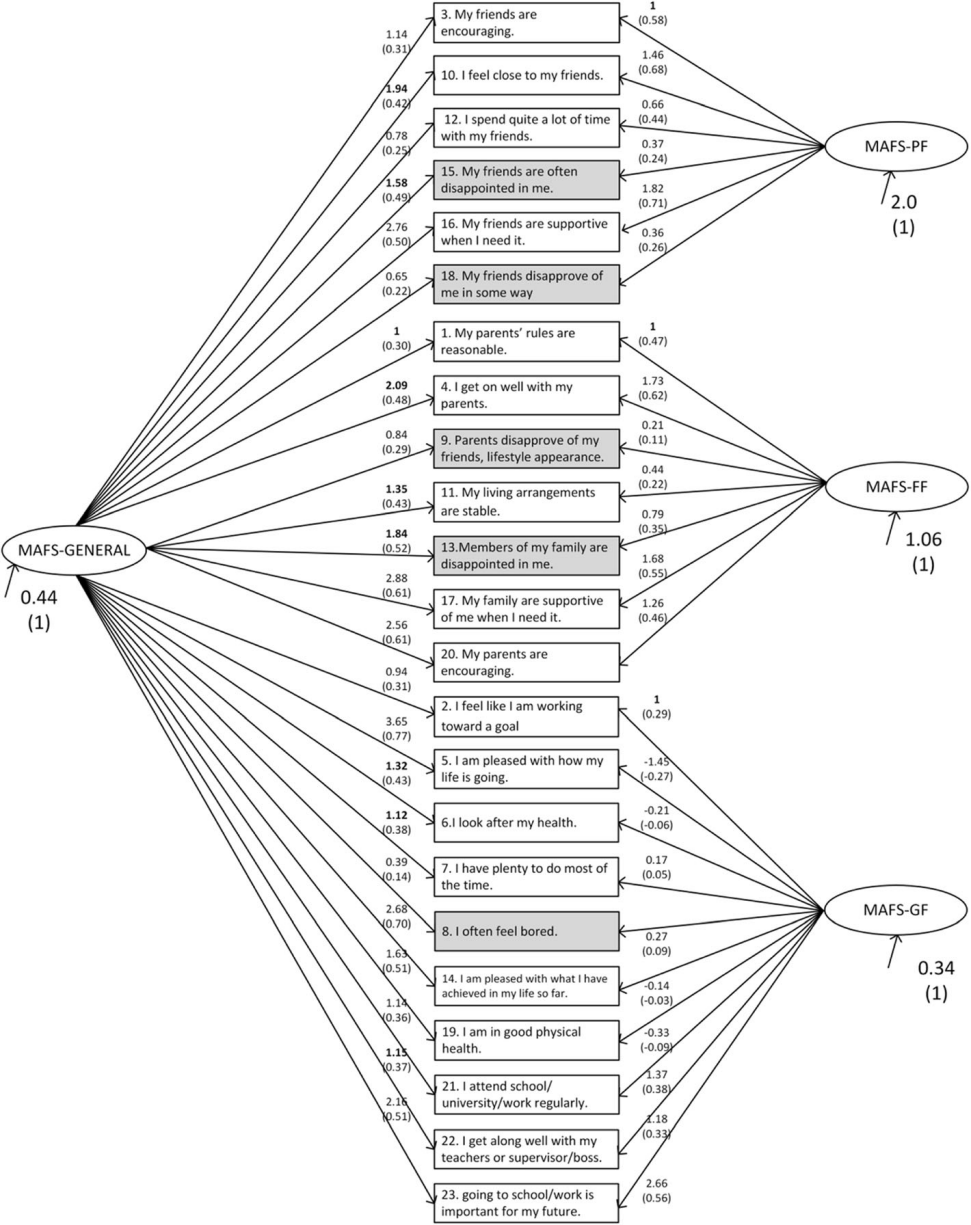
The best-fitting bifactor model in the confirmatory factor analyses. All unstandardized and standardized (between parentheses) parameters are presented. Reverse-scored items are shaded grey. Fixed parameters (first item loading per factor) printed in bold font

CFA results are shown in Table 2. CFA based on MLR showed that the BIC was lowest for the 3-factor model and the AIC was lowest for the bifactor model. Additional CFAs using the WLSMV estimator showed that the CFI was highest and the RMSEA was lowest for the bifactor model. Based on these results, the bifactor model was taken to best represent the MAFS data (Fig. 1). Additional CFAs that addressed a potential method effect by freely estimating residuals among reverse-score items, showed that this increased model fit for all models, including the bifactor model.

**Table 2:** Confirmatory Factor Analyses with the MAFS data (*n* = 397)

	MLR estimation (FIML)				WLSMV estimation^b^ (pairwise)		
	Number of free parameters	Log likelihood	AIC	BIC	Chi-square (df)	CFI	RMSEA
1-factor model	69	− 7135.6	14,409.1	14,684.0	440.1 (93)*	0.71	0.097
1-factor model (M)^1^	79	–	–	–	430.2 (91)*	0.72	0.097
3-factor model	72	− 7004.7	14,153.4	14,440.3	262.3 (95)*	0.86	0.067
3-factor model (M)^1^	82	–	–	–	243.3 (93)*	0.88	0.064
Bifactor model	92	− 6970.3	14,124.6	14,491.1	215.7 (89)*	0.90	0.060
Bifactor model (M)^a^	102	–	–	–	197.4 (88)*	0.91	0.056

^a^In these models, the residuals of the reverse-scored items are set to be freely estimated to account for a possible method effect

^b^WLSMV bases model estimation on a pairwise estimated polychoric correlation matrix, which is less optimal than

*p < 0.001

FIML in case of missing values. It is merely included to obtain the CFI and RMSEA

*MLR* Robust maximum likelihood, *FIML* Full information maximum likelihood, *WLSMV* Mean and variance adjusted weighted least squares, *AIC* Akaike Information Criterion, *BIC* Bayesian Information Criterion, *CFI* Comparative Fit Index, *RMSEA* Root Mean Square Error of Approximation

### Factor associations with demographics and other measures

Mean factor scores of the bifactor model in different gender and education-level groups (Table 5 in Appendix**)** showed that the mean PF score was significantly higher in females compared to males and decreased with education level. The mean FF factor score increased with education-level.

Correlations of the factors of the bifactor model with age and other administered questionnaires (Table 3) showed that the general factor was associated with all variables except sick days from school. Several constructs (i.e., SDQ difficulties, WHO Total and school grades) were only associated with this general factor. In addition, diverging patterns were seen in that the PF factor was associated with the GVSG Friends scale and the FF factor with the GVSG Parents scale. Happiness was associated with the two most general factors underlying the MAFS (the General factor and the GF factor) and the SDQ Prosocial score was associated with all other measures except the FF factor. Correlations of MAFS scale sum scores with age and the other questionnaires are provided in Table 6 in Appendix.

**Table 3:** Correlations (Spearman’s Rho) of factor scores with age and other psychometric instruments

	PF factor	FF factor	GF factor	General factor
Age	0.10	**−0.17**	**0.14**	**−0.18**
SDQ Difficulties	− 0.04	− 0.07	− 0.03	**−0.47**
SDQ Prosocial	**0.25**	0.04	**0.20**	**0.24**
GVSG Parents	0.05	**0.38**	0.02	**0.47**
GVSG Friends	**0.51**	−0.02	0.07	**0.34**
WHO total	0.03	0.07	−0.04	**0.54**
Happiness	−0.07	0.10	**−0.26**	**0.52**
School grade	−0.04	0.09	0.04	**0.33**
Sick days	0.04	−0.05	**0.14**	−0.07

*GVSG* Groningse Vragenlijst Sociaal Gedrag, *SDQ* Strengths and Difficulties Questionnaire, *WHO* WHO Well-being Index

Coefficients printed in bold font are statistically significant (*p* < 0.05)

### Scale correlations and internal consistency

Computed from the raw data, the three subscales showed significant correlations (Table 4), with patterns of correlations being similar for males and females. Internal consistency of the MAFS-TS was good (α = 0.87). The internal consistencies of the three subscales were acceptable (α = 0.74–0.80). Comparable alphas were found in males and females.

**Table 4:** Scale correlations (Spearman’s Rho) and internal consistency coefficients (Alpha)

Sample	Scale	MAFS General	MAFS Family	MAFS Peer	MAFS Total
sample	MAFS Total	–	–	–	**0.87**
	MAFS General	**0.74**	–	–	–
	MAFS Family	0.43^a^	**0.80**	–	–
	MAFS Peer	0.36^b^	0.33^a^	**0.77**	–
Male	MAFS Total	–	–	–	**0.87**
	MAFS General	**0.72**	–	–	–
	MAFS Family	0.42^a^	**0.80**	–	–
	MAFS Peer	0.40^b^	0.25^a^	**0.76**	–
Female	MAFS Total	–	–	–	**0.88**
	MAFS General	**0.76**	–	–	–
	MAFS Family	0.44^a^	**0.81**	–	–
	MAFS Peer	0.34^b^	0.37^a^	**0.77**	–

*MAFS* Multidimensional Adolescent Functioning Scale. All correlations significant at *p* < 0.01

Cronbach’s alpha coefficients were computed based on polychoric item correlation matrix computed in the total sample on pairwise complete observations

^a^Computed for participants with complete observations: n = 335 (total), *n* = 116 (male sample), *n* = 219 (female sample)

^b^Computed for participants with complete observations: *n* = 354 (total), *n* = 120 (male sample), *n* = 234 (female sample)

## Discussion

**Fig. 2:**
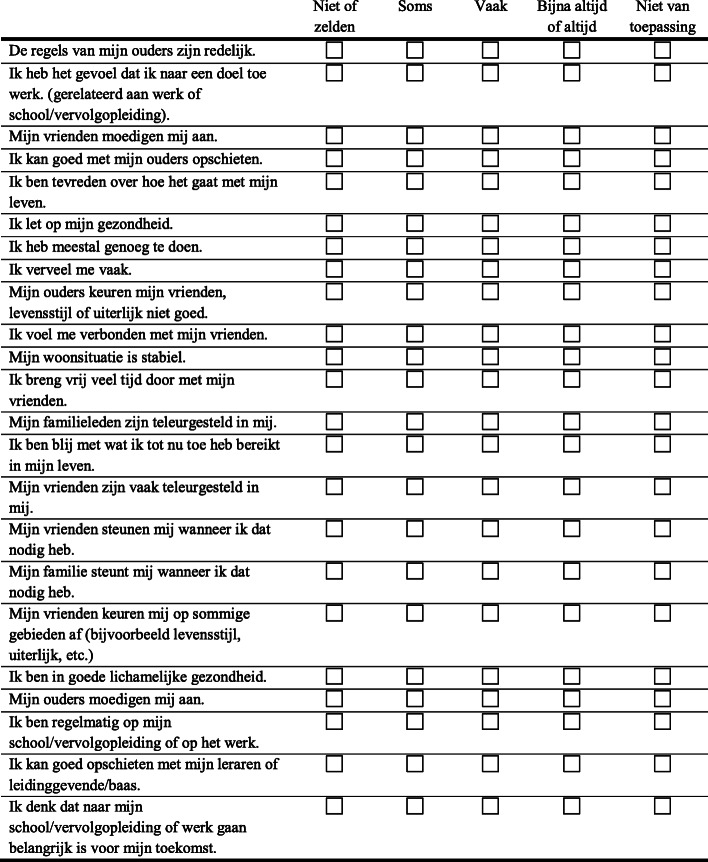
Dutch translation of the MAFS

In this study, we aimed to investigate the psychometric properties of the Dutch translation of the Multidimensional Adolescent Functioning Scale (MAFS). A hierarchical/bifactor model with four uncorrelated factors: a general factor loading on all items (MAFS-general) and three group factors, loading respectively on the General Functioning, Family Functioning and Peer Functioning items, was found to describe the data best. These three group factors align with the three subscales of the MAFS. Patterns of convergent and divergent validity were as expected, with positive correlations between MAFS factor scores and other measures of functioning, and negative correlations between MAFS factor scores and measures of dysfunction. In addition, different patterns of correlations between the latent factors and related measurements provided evidence for differentiation between the different domains covered by the MAFS in terms of their content coverage, supporting the convergent and divergent validity of the scales. Correlations between raw scores of the three subscales were significant, but low enough to support the subscales’ measurement of differentiated constructs. Internal consistency of the MAFS Total score was good and internal consistencies of the three subscales were acceptable. Together, these results indicate that the translation of the MAFS to the Dutch language has resulted in a measure with adequate psychometric properties that are comparable to those of the original English language version. The questionnaire is freely available (Figure 2 in Appendix) and can be used without restrictions for non-commercial purposes.

Three group factors were found that align with the original MAFS subscales; however, the addition of an uncorrelated general factor was found to improve the model fit in the current sample. This latter factor explains all variance that the items share in common and can be interpreted as reflective of the general underlying construct of general functioning. The significant correlations of the general factor with all other questionnaires that assess functioning and psychopathology (except sick days) supports this interpretation. Interestingly, several other measures that also reflect more general constructs (i.e., SDQ Difficulties score, WHO Total score and school grades) are correlated *only* with this general factor, suggesting that these are not differentially associated with certain sub-domains of functioning but tap into a broader level of functioning. The group-factors explain additional variance that is shared in common by subsets of items. The behaviour of the three group factors in terms of associations with the other measures also follow patterns that align with the initial design of the instrument, especially the finding that the PF factor is only associated with another measure of functioning in the context of peers (GVGS Friends subscale) and the FF factor only with another measure of functioning in the context of family (GVSG Parents subscale). These findings support the idea that the MAFS can capture both the general adolescent functioning level (by using the total score), as well as functioning in different domains (by using the subscale scores).

Overall, the currently found psychometric properties of the Dutch version of the MAFS are comparable to the psychometric properties that were reported for the original English version [2]. The factor model fit in the current study was lower than in the original study (although still acceptable); there are several potential reasons for this. First, the sample in the original study was more than twice as large as the current sample and included mostly 15–16 year olds, which may have influenced the estimates. Second, it could be that the subdomains are associated differently with each other or that some items of the questionnaire behave differently in different populations, e.g., with different cultural backgrounds. For example, people from different cultures may differ in the way family needs or individual needs are prioritized. Also, cultures may differ in the extent to which autonomy of the adolescent is encouraged by parents or to which parental authority is respected by children [23]. For example, the item, ‘my parents’ rules are reasonable’ may be answered very differently by an adolescent with high respect for parental authority than by an adolescent with high value for autonomy. The sample of the original validation of the MAFS is likely more heterogeneous than the current sample in terms of e.g., age and cultural background and this may be a potential explanation for the observed differences. Some final considerations should be kept in mind with regard to the factor-analytical results: first, the final models in two studies are not directly comparable. The previous study only tested a 3-factor model and no bifactor model. However, the fact that all other properties were comparable suggests relative stability of the questionnaire’s properties across the two versions. Finally, the fit of the best, bifactor model in the current study was ‘acceptable’ according to the CFI and RMSEA, but not very good. To obtain better fit, further fine-tuning of the model could have been undertaken. However, the downside of such an approach would have been that the eventual model would likely be over-fitted to our specific data and its specific random variations and outliers, limiting generalizability. We therefore did not seek further for a ‘better’ model.

One point of particular interest is the response category ‘not applicable’ that is available for each individual item in the MAFS. Several individuals checked this option for some questions of a subscale but not for all, making it unclear whether this means that an item is not applicable only for a specific item (e.g., not being able to talk to your mother about problems because you never talk with your mother but you might do other things together) or because the person that is referred to in the item is absent (i.e. not being able to talk to your mother because you have no mother figure present). If there is no mother figure, none of the items pertaining to contact with a mother can be completed. However, if there is a mother figure but the adolescent does not have in depth conversations with her, then the NA response could be given here, but not on other questions about the mother. Therefore, to improve interpretability, we recommend careful consideration of this response category. One suggestion for future use could be adding more general descriptive terms to the items, for example adding “a parent, guardian, mentor or anyone else close to you” to the items pertaining to father or mother figure. The same could be considered for friends (e.g., friend, acquaintance, peer). Also, consideration could be given to the instructions provided to respondents on this category, especially in lower levels of education, or how to deal afterwards with responses given (e.g., recoding, excluding per item or per subscale, etc.).

The current study has several limitations. First, only internal consistency of the scales were assessed, whereas test-retest reliability would have provided insight into the true reliability of the scales. Wardenaar and colleagues [2] also investigated the stability of the MAFS over time with a baseline, 1-year and 3-year follow-up and found similar psychometric properties at all three time points. We expect that this will also be applicable to the Dutch version of the MAFS, but future research will need to confirm this. Second, the majority of our participants were in the older age range of the intended target group (i.e., adolescents between 12 and 17 years old); however, as younger individuals were even more underrepresented in the original validation study, our study can be seen as complementing the original investigation. Third, presence of mental or somatic disorder was not assessed in the study. When applied to clinical adolescent populations, the psychometric properties of the questionnaire might differ. Fourth, although utmost care was taken in the translation procedures, we did not include members of the target population (i.e. adolescents) in the translation team, which could have helped to further boost the quality and usefulness of the translated MAFS, including taking into account potential differences in (cultural) background. Finally, our selection strategy has some consequences. For this study, we selected adolescents based on age and school level. This has resulted in a sample in which, in terms of demographic information, each group (gender, age, educational level) is represented. However, the distribution of the sample in terms of these characteristics is not representative of the total adolescent general population and therefore, generalizability of the results is limited. Specifically, our findings might be less applicable to adolescents under 16 years old. In our sample, 59.6% of the adolescents were 16–17 years and thus, younger adolescents were underrepresented. Due to relatively small sample size, contingency tables used to estimate polychoric correlations for the confirmatory factor analysis with WLSMV contained zero frequency cells [24]. This made the estimations of the polychoric correlations difficult and thus the results of the factor analysis less reliable. Lastly, there could be potential biases in our study. Approximately 20% of the approached adolescents did not participate in our study due to various reasons,(e.g., they or their parents did not consent or the adolescent forgot to bring their signed informed consent form to school during the assessment), which may have led to some degree of selection bias, making the results less generalizable to all adolescents in the population. Although a small part (19.6%) of the surveys were collected by school teachers, teachers were instructed carefully and therefore we estimate a potential instruction bias to be limited.

Future research could evaluate a broader range of psychometric characteristics, especially in younger (12–14 years) adolescents and could investigate the predictive value of the MAFS for later physical and mental health problems. The latter is of specific interest, as earlier research stated that subtle functional changes in healthy adolescents could predict future psychiatric or physical illnesses [7, 8, 25]. Henceforth, replicating such studies with the MAFS in the Netherlands might be informative. In addition, the factor structure of the MAFS could be further investigated, in particular, the role and impact of the NA response category. Finally, many interesting insights into convergent/divergent validity and the latent structure of the MAFS could in theory be gained by inclusion of covariates directly in the CFA model. Running such Multiple Indicator Multiple Cause (MIMIC) models are, however, complicated by the fact that their application to bifactor/hierarchical factor models is not straightforward [26].

## Conclusions

The Dutch version of the MAFS has good psychometric properties that in general are comparable to its original English version. As such, the scale can be used to validly, reliably and easily as a measure of functioning among adolescents from the general population in the Netherlands. It could provide professionals working with adolescents with a useful and easy-implementable screening tool. The Dutch translation of the MAFS can be found in Figure 2 in Appendix and is freely available for use.

## Data Availability

Data are available upon reasonable request from the corresponding author.

## Appendix

**Table 5:** MAFS bifactor model factor scores for different gender and education-level groups

	MAFS_GEN		MAFS-PF		MAFS-FF		MAFS-GF	
	mean	sd	mean	sd	mean	sd	mean	sd
male	−0.04	0.60	−0.35	1.09	−0.03	0.73	− 0.03	0.73
female	0.01	0.58	0.18	1.12	0.01	0.71	0.02	0.71
	F = 0.66; *p* = 0.42		F = 20.1; P < 0.001		F = 0.23; *P* = 0.63		F = 1.32; *p* = 0.25	
low education	−0.06	0.57	0.28	1.13	−0.20	0.81	0.08	0.41
mid education	−0.08	0.58	0.03	1.18	−0.02	0.69	−0.05	0.38
high education	0.04	0.6	−0.10	1.12	0.07	0.68	−0.01	0.38
	F = 2.46; *p* = 0.12		F = 6.78; *P* = 0.01		F = 8.34; *p* = 0.004		F = 1.90; *p* = 0.17	

**Table 6:** Correlations of the MAFS scales with other measures

Sample	External measures	MAFS Total	MAFS General	MAFS Family	MAFS Peer
Total	SDQ Difficulties	−0.45	− 0.41	−0.34	− 0.27
	SDQ Prosocial	0.33	0.29	0.23	0.27
	GVSG Parents	0.50	0.42	0.53	0.25
	GVSG Friends	0.48	0.30	0.22	0.57
	WHO total	0.52	0.50	0.38	0.26
	Happiness	0.42	0.40	0.38	0.16
	School grade	0.31	0.28	0.25	0.12
	Sick days	−0.01^n.s.^	−0.07^n.s.^	−0.06^n.s.^	0.04^n.s.^
Male	SDQ Difficulties	−0.42	−0.34	− 0.29	−0.30
	SDQ Prosocial	0.39	0.33	0.39	0.23
	GVSG Parents	0.47	0.39	0.47	0.23
	GVSG Friends	0.48	0.35	0.20	0.58
	WHO total	0.48	0.49	0.33	0.20
	Happiness	0.44	0.41	0.36	0.21
	School grade	0.31	0.28	0.24	0.13^n.s.^
	Sick days	0.10^n.s.^	0.05^n.s.^	−0.05^n.s.^	0.11^n.s.^
Female	SDQ Difficulties	−0.48	−0.45	− 0.38	−0.28
	SDQ Prosocial	0.27	0.26	0.15	0.22
	GVSG Parents	0.50	0.43	0.55	0.26
	GVSG Friends	0.45	0.27	0.20	0.53
	WHO total	0.57	0.51	0.42	0.35
	Happiness	0.45	0.41	0.40	0.19
	School grade	0.30	0.28	0.26	0.09^n.s.^
	Sick days	−0.08^n.s.^	−0.13	−0.07^n.s.^	−0.03^n.s.^

MAFS = Multidimensional Adolescent Functioning Scale, GVSG = Groningse Vragenlijst Sociaal Gedrag, SDQ = Strengths and Difficulties Questionnaire, WHO = WHO Well-being Index

^n.s.^ Non-significant correlations. All other correlations significant at p < 0.05
